# Tools and tutorial on practical ray tracing for microscopy

**DOI:** 10.1117/1.NPh.8.1.010801

**Published:** 2021-01-21

**Authors:** Valérie Pineau Noël, Shadi Masoumi, Elahe Parham, Gabriel Genest, Ludovick Bégin, Marc-André Vigneault, Daniel C. Côté

**Affiliations:** a Université Laval, CERVO Brain Research Center, Québec, Canada; b Université Laval, Centre D’Optique, Photonique et Laser, Québec, Canada

**Keywords:** coding, optical engineering, imaging systems, illumination

## Abstract

**Significance:** An advanced understanding of optical design is necessary to create optimal systems but this is rarely taught as part of general curriculum. Compounded by the fact that professional optical design software tools have a prohibitive learning curve, this means that neither knowledge nor tools are easily accessible.

**Aim:** In this tutorial, we introduce a raytracing module for Python, originally developed for teaching optics with ray matrices, to simplify the design and optimization of optical systems.

**Approach:** This module is developed for ray matrix calculations in Python. Many important concepts of optical design that are often poorly understood such as apertures, aperture stops, and field stops are illustrated.

**Results:** The module is explained with examples in real systems with collection efficiency, vignetting, and intensity profiles. Also, the optical invariant, an important benchmark property for optical systems, is used to characterize an optical system.

**Conclusions:** This raytracing Python module will help improve the reader’s understanding of optics and also help them design optimal systems.

## 1 Introduction

Engineers and scientists operating in fields such as neurophotonics, remote sensing, medicine, or even industrial manufacturing are often tasked with building or modifying imaging systems for customized applications. They must often resort to expert advice from optical designers to obtain clear and quantitative answers to their specific optics problems. Optical designers, with their knowledge and specialized software tools such as Zemax, Oslo, or CODE V, can indeed provide these answers. However, third-party expert advice can be prohibitively expensive, difficult to interpret without basic knowledge, and can lead to sub-optimal solutions if the requirements are miscommunicated to the external consultant. For the general engineer, scientist, and especially for trainees, learning these professional software tools is often not an option because of the abundance of features targeted to advanced optical designers that end up confusing trainees and non-experts alike. Yet, there should be a solution, between simplified analytical solutions and expert tools that can help non-experts tackle moderately complicated problems by themselves to obtain quantitative answers. It is therefore the purpose of the present tutorial to provide both the knowledge and the tools for this audience.

In this paper, we describe both optical design concepts and a raytracing module based on the ray matrix formalism (i.e., ABCD matrices) to characterize important aspects of real optical systems typically encountered by non-experts, such as the effect of finite-sized lenses and apertures. The motivation for this project and tutorial was borne out of teaching a second-year optics class where it became clear that students understood the formalism but were not always able to obtain quantitative answers to practical problems for the laboratory. This led to the creation of the present module to explain the calculations and to provide a straightforward method to characterize reasonably complex optical systems. Over its development cycle, it has found a niche in research labs when professional tools are not available. There are two guiding principles: simplicity of usage and clarity of implementation for teaching purposes. The module, therefore, aims to model and evaluate optical systems, characterize their properties, and identify their weaknesses from the perspective of a non-expert optical designer. As will be shown, the module is specifically designed to assess microscopes but can also be used to evaluate fiber-based devices or illumination devices.

The outline of the present tutorial is therefore as follows. Sec. 1 presents the installation of the raytracing module with its documentation and is followed by a brief description of the ray matrix formalism in Sec. 3. Section 4 describes how to use the formalism to consider the finite size of elements and identify the limiting components of any optical system. Then, Sec. 5 dives into the module and rapidly presents some of its main functionalities. Finally, Sec. 6 shows practical examples that should be of interest to the readers. This tutorial ends with a discussion and a brief conclusion at Secs. 7 and 8, respectively.

## 2 Raytracing Module

The raytracing module is programmed in Python for several reasons. First, Python is widely available and used in science by beginners and experts alike. In addition, the ray-tracing formalism is particularly well suited to object-oriented software design, and the Python language offers very good support for such paradigm. Third, the Python community has developed many tools to support development, such as a straightforward and powerful documentation system from within the code when needed (docstring, Sphynx, readthedocs, and unit testing) and also simple distribution tools (GitHub and PyPI). Finally, while Python is an interpreted language (which simplifies development), it offers a reasonably high performance for scientific calculations (which favors its adoption).

One can install (or upgrade) the raytracing module with the following in a Python command prompt:

pip install raytracing --upgrade

and learn the basics of this module by referring to the examples in Secs. 5 and 6. A more detailed installation procedure is available^1^ including videos if needed.^2^ Finally, documentation that describes all the elements, functions, and details of this module is available online at Ref. 3. The code is open source. We have followed the guidelines from Clean Code:^4^ clear implementation over high-performance implementation, documentation within the code when needed, and unit testing.

## 3 Formalism

### 3.1 Rays through Optical Elements

For completeness, we start with a short summary of the ray matrix formalism that describes the propagation of a ray along an optical axis ($\hat{z}$) and how it is transformed by matrices representing optical elements. A ray at a certain position z along the optical path is defined by a column vector as

$$r\equiv [\begin{array}{c} y\\ \theta \end{array}],$$ (1)

where y is the height of the ray with respect to the optical axis and θ is the angle that this ray makes with the optical axis. It is worth noting that there are other definitions of the ray^5^ that include the index of refraction directly in the ray definition, but these are not used here. In the raytracing module, a ray will be an instance of Ray, and a collection of rays will be an instance of Rays.

The ray matrix formalism allows a ray to be transformed from one reference plane to another through different optical elements, all represented by 2×2 matrices of the form:

$$M=[\begin{array}{cc} A & B\\ C & D \end{array}]$$ (2)

**Fig. 1:**
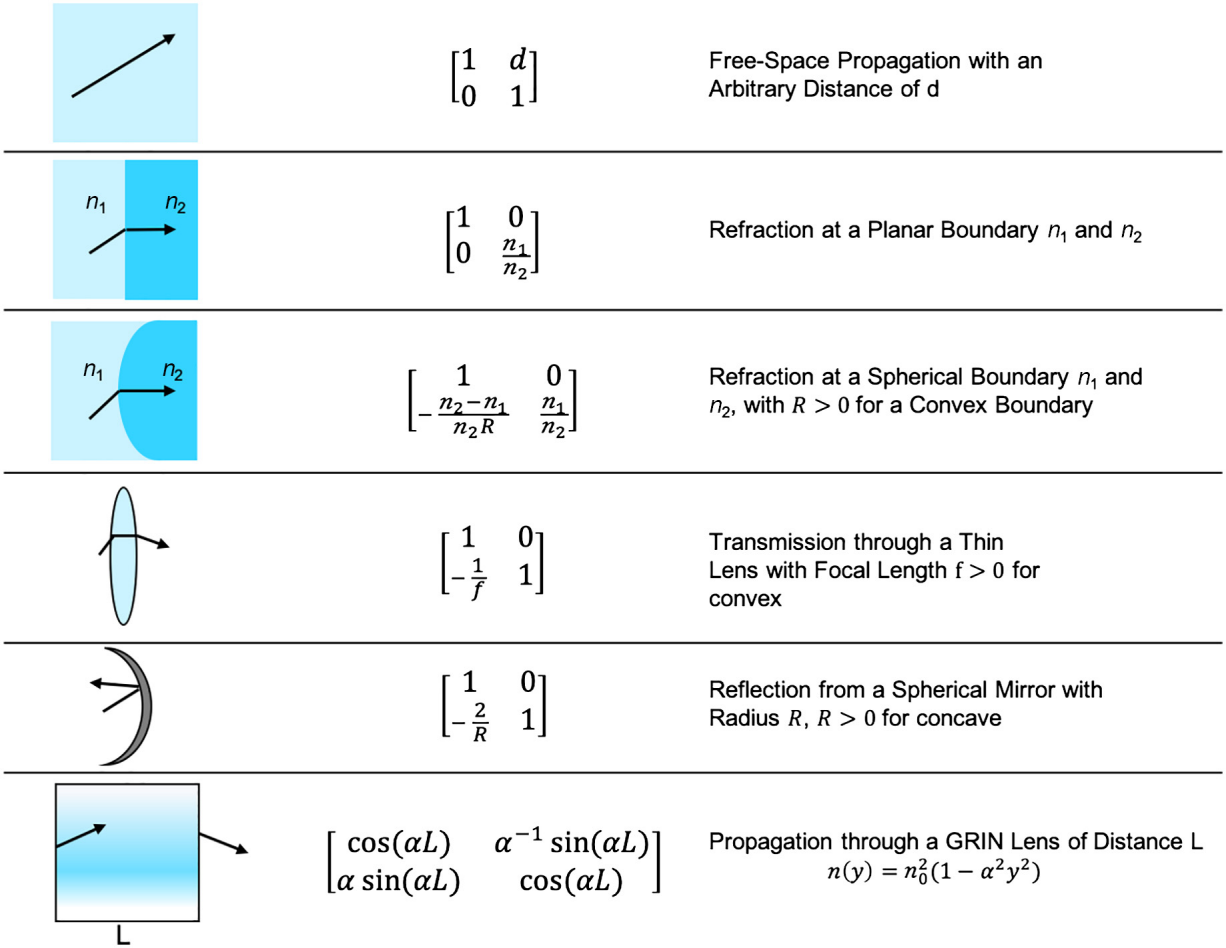
ABCD matrices of different optical elements within the paraxial approximation.^6^

Such a matrix transforms a ray with a left multiplication: a ray r that $\left(M_{1},M_{2},M_{3},\ldots ,M_{i}\right)$ will be transformed into $r^{\prime }$ by the sequential left application of the matrices representing the sequence of elements (note the order of multiplication):

$$r^{\prime }=M_{i},\ldots M_{3}M_{2}M_{1}r=\mathrm{Mr},$$ (3)

which results in

$$y^{\prime }=Ay+B\theta ,$$ (4)

$$\theta^{\prime }=Cy+D\theta ,$$ (5)

where A, B, C, and D are the elements of the transfer matrix and the necessary paraxial approximation, i.e., sin θ≈θ, is used: the consequences of foregoing the paraxial approximation would be significant and will be addressed in the discussion. The exact form of each matrix representing optical elements is obtained using Snell law and geometrical considerations.^6^ A list of the most important ray matrices is shown in Fig. 1. The optical system is modeled by appending the desired matrices (e.g., Space, Lens, System4f) to an ImagingPath (see Sec. 5 for more details).

### 3.2 Useful Properties

**Fig. 2:**
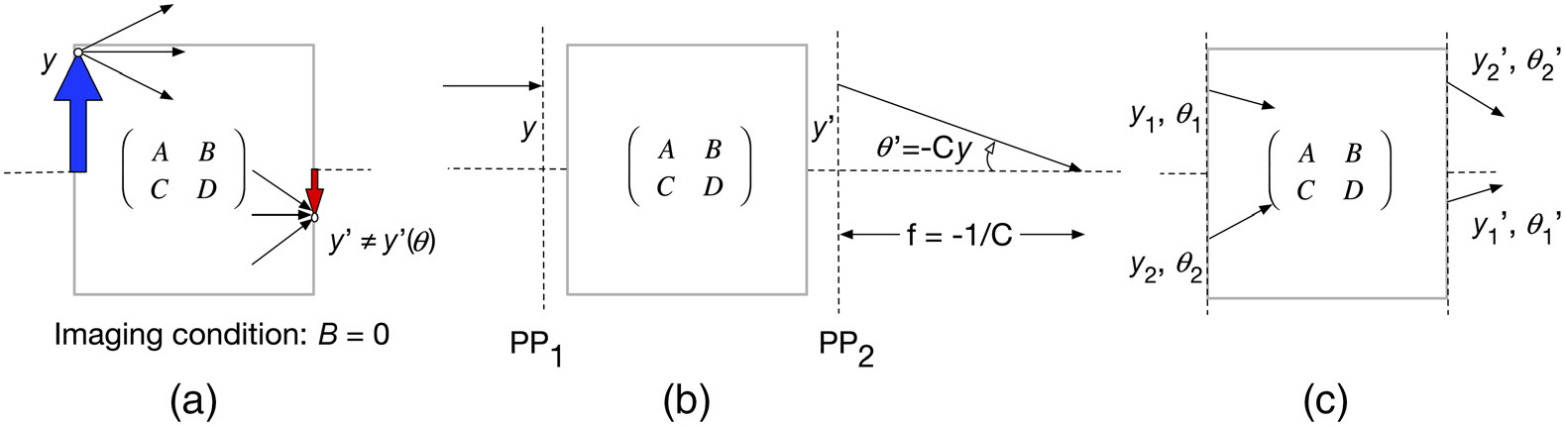
Illustration of a ray passing through an optical system defined by a generic ABCD matrix, dotted line is the optical axis. (a) The object (blue arrow) is imaged (red arrow) on the other side of the optical system, i.e., to its conjugate plane. (b) A ray parallel to the optical axis will go through the focal plane. (c) The input ray height y and angle θ have changed to $y^{\prime }$ and $\theta^{\prime }$, respectively, according to the ABCD matrix. The product $n(y\theta^{\prime }-y^{\prime }\theta )$ is constant everywhere in the system.

With this knowledge of the formalism, important properties can be extracted for any optical system:

1. When B=0, we have an imaging condition where an object at the entrance is imaged at the exit plane since a ray originating from a height y reaches a height that is independent of the angle of emission, i.e., $y^{\prime }=Ay$ not $y^{\prime }=Ay+B\theta$. Naturally, in this situation, A is the transverse magnification and D is the angular magnification [Fig. 2(a)]. This property of an element or a group of elements can be verified in an imaging path with this module using isImaging.
2. The effective focal distance at the back of any system is $C=-\frac{1}{f}$ [Fig. 2(b)], and it can be obtained for any element with effectiveFocalLengths. In general, the front and back focal lengths (BFLs) are different if the media on either side are different, but in many cases of interest, they will be identical.
3. Focal distances are measured from principal planes, which are planes of unity magnification in any system where all the focusing power is concentrated. They are located $L_{{\mathrm{PP}}_{f}}=\frac{n_{1}/n_{2}-D}{C}$ in front, and $L_{{\mathrm{PP}}_{b}}=\frac{1-A}{C}$ after the input and output reference planes [Fig. 2(b)]. The position of the two principal planes of any components can be obtained using principalPlanePositions in the module.
4. Finally, it can be shown that for any two rays i and j, the following quantity
  $$I_{ij}=n(y_{i}\theta_{j}-y_{j}\theta_{i}),$$ (6)
  at a given point is a constant throughout the system and is called the optical invariant [Fig. 2(c)]. It can be calculated in a system with opticalInvariant(ray1,ray2) for any two rays (more details in Sec. 5). When the principal and axial rays are used (defined below), the optical invariant is maximal and is rather called the Lagrange invariant noted by H, obtainable with lagrangeInvariant().^7^:

It is already possible to obtain, or rediscover, interesting results. For instance, it is fairly straightforward to obtain Gauss’s law of imaging by modeling an object $d_{o}$ in front of a thin lens of focal f: the position of the image is obtained when the transfer matrix that includes the propagation by a distance $d_{i}$ after the lens has B=0. Another example is the Lensmaker equation that describes the focal distance of a lens using the radii of the two curved surfaces and the index of refraction of the material: one can model the interfaces and the material, then obtain the focal distance with C=−1/f. There are many other examples that will not be discussed in detail here, but one can read more about them if needed.^6^^,^^8^^,^^9^^,^^10^ It can therefore be appreciated that despite its simplicity, the formalism recovers all known results from paraxial optics and will be sufficient to model most optical systems. The only aspect we have not considered yet is the effect of apertures that can block rays: this is considered in the next section.

## 4 Apertures

The ray matrix formalism is surprisingly complete and sufficient for many tasks. However, it does not consider the single most important limitation of real instrumentation: the finite sizes of elements or aperture. To consider the finite size of the optics in ray propagation, the height of the rays must be checked at each point along the propagation axis to make sure it is not blocked. To do so, tracing the rays one by one, i.e., ray tracing is necessary to identify those limiting optics, which can be done with trace() or traceThrough(), or other similar functions. Using this tracing procedure enables the identification of two essential apertures characterizing an optical system, the aperture stop (AS), and the field stop (FS).

**Fig. 3:**
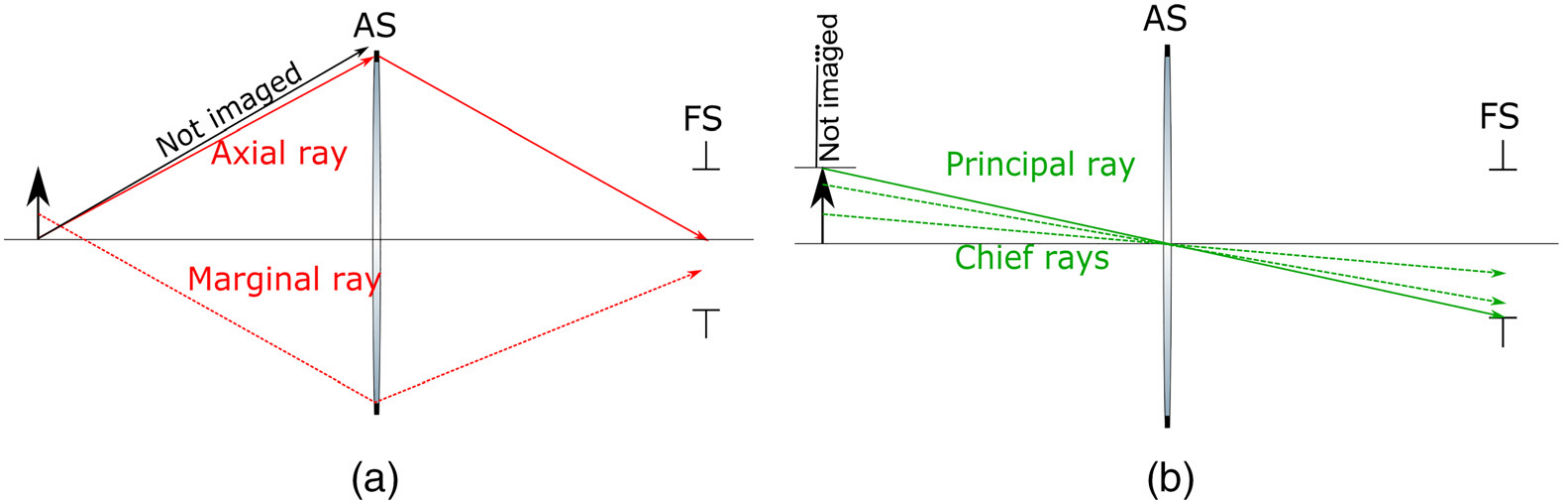
Simple system made of a lens and an aperture presenting the propagation of different rays. (a) presents the axial and marginal rays. The axial ray has the highest possible angle. Thus, any other ray with a greater angle cannot make it to the image plane (solid dark arrow). (b) presents the principal ray and the chief ray. Any ray starting with a greater height than the principal ray hits FS and therefore is not included in the image formation.

The AS is the physical aperture that limits the acceptance cone of light entering the system from a point on axis.^8^ Hence, a properly located AS will maximize the light collection and a misplaced or improperly sized AS will reduce it. Any ray from the object that hits the edge of AS is called a marginal ray, and the unique ray originating on axis that hits the edge of AS is called the axial ray.^11^ Many authors^12^^,^^13^ define this ray as the marginal ray, but in this paper, this term is rather employed to define any ray passing at the maximal possible height of AS, regardless of the starting point on the object. All of these rays are shown in Fig. 3(a). To find the AS in an optical system, one can use the ABCD matrices to find the height r(z) of a ray at all positions z, then divide it by the real diameter D(z) at z. The AS location is where the ratio $\frac{r(z)}{D(z)}$ has the highest value.^6^ In the module, the position and diameter of the AS in an imaging path can be obtained using apertureStop(). In addition, two functions are defined to obtain the axial and marginal rays (i.e., their heights and angles) with axialRay() and marginalRays(), respectively.

Second, the FS is the physical aperture that limits the field-of-view (FOV, at the object) and the image size (at the image). To find the location of the FS, one traces a ray from different heights passing through the center of the AS until it gets blocked by an aperture, which is labeled the FS. The position and the diameter of FS can be found using fieldStop() in an imaging path. It should normally be the finite size of the sensor located at the camera,^8^ as presented in Figs. 3(a) and 3(b). When FS appears anywhere except at the camera, it will produce vignetting on the final image, which is the presence of dark blur, indicating that part of the cone of light from a source point does not reach the sensor. Even if the FS is at the image plane, vignetting can still occur if the diameter of a lens in the system is too small^12^ (see example Sec. 6.3). The presence of vignetting in a system is the most common error by trainees and can readily be identified with the module.

Any ray from the object going through the center of AS is called a chief ray, and the last chief ray that is not blocked by FS is called the principal ray,^11^ as shown in Fig. 3(b). While some authors use the term chief ray instead of principal ray,^12^^,^^13^ it is used in this paper to define any ray still passing through the center of AS but starting at any point on the object [Fig. 3(b)]. More specifically, the origin of the principal ray defines the field of view. Both of these rays can be obtained with chiefRay() and principalRay(). It is worth noting that AS position in an optical system must be known to find FS. In closing this section on the formalism, it is interesting to know that the principal and axial rays are the only two rays needed to completely characterize an optical system: any ray can be expressed as a linear combination of these two rays (see Sec. 6.3 where this is used to discuss efficiency). As can be appreciated, finding the AS and FS can become tedious if done manually, however, it is perfectly adapted to a computer program. We will now dive into the module and illustrate with examples how to obtain all optical properties dependent on AS and FS, which is critical to determine the real-life performance of any optical system.

## 5 Module Overview

The Python raytracing module is an implementation of the ray matrix formalism discussed previously. This section introduces its main features and functionalities. Specific functions from the module are typed in monospace fonts such as System4f(). Complete code and documentation are available online.^3^

### 5.1 Creation of an Optical Path

The first step is to create an optical path using ImagingPath(). Many elements, such as Lens(), Space(), Aperture(), and more, are defined and can be added to the imaging path using append(). As an example use, a simple 4f relay is presented in the following to show how to create an imaging path in raytracing and how to add elements.

**from** raytracing **import** *

path = ImagingPath()

path.label="Simple example"

path.append(Space(d=50))

path.append(Lens(f=50, diameter=25, label="First lens"))

path.append(Space(d=100))

path.append(Lens(f=50, diameter=25, label="Second lens"))

path.append(Space(d=50))

*# For convenience, the creation of a 4f relay can be written as:*

*# path.append(System4f(f1=50, f2=50, diameter1=25, diameter2=25))*

*# Lenses from vendors can be added with:*

*# path.append(olympus.XLUMPlanFLN20X())*

*# path.append(thorlabs.AC254_100_A())*

path.display()

Many optical elements are already incorporated in the module and can therefore be included in any imaging path. For example, *olympus.py* contains many objectives from Olympus and *thorlabs.py* has achromatic lenses from Thorlabs. Similarly, for convenience System2f() for propagation focus-to-focus and System4f() for a 4f-relay are defined in the module and used in the remaining of this tutorial.

As for the display function, it depicts the path and the traced rays starting at the object plane via an interface, where either rays from an object or the principal and axial rays can be shown as it is discussed next.

### 5.2 Visualization

**Fig. 4:**
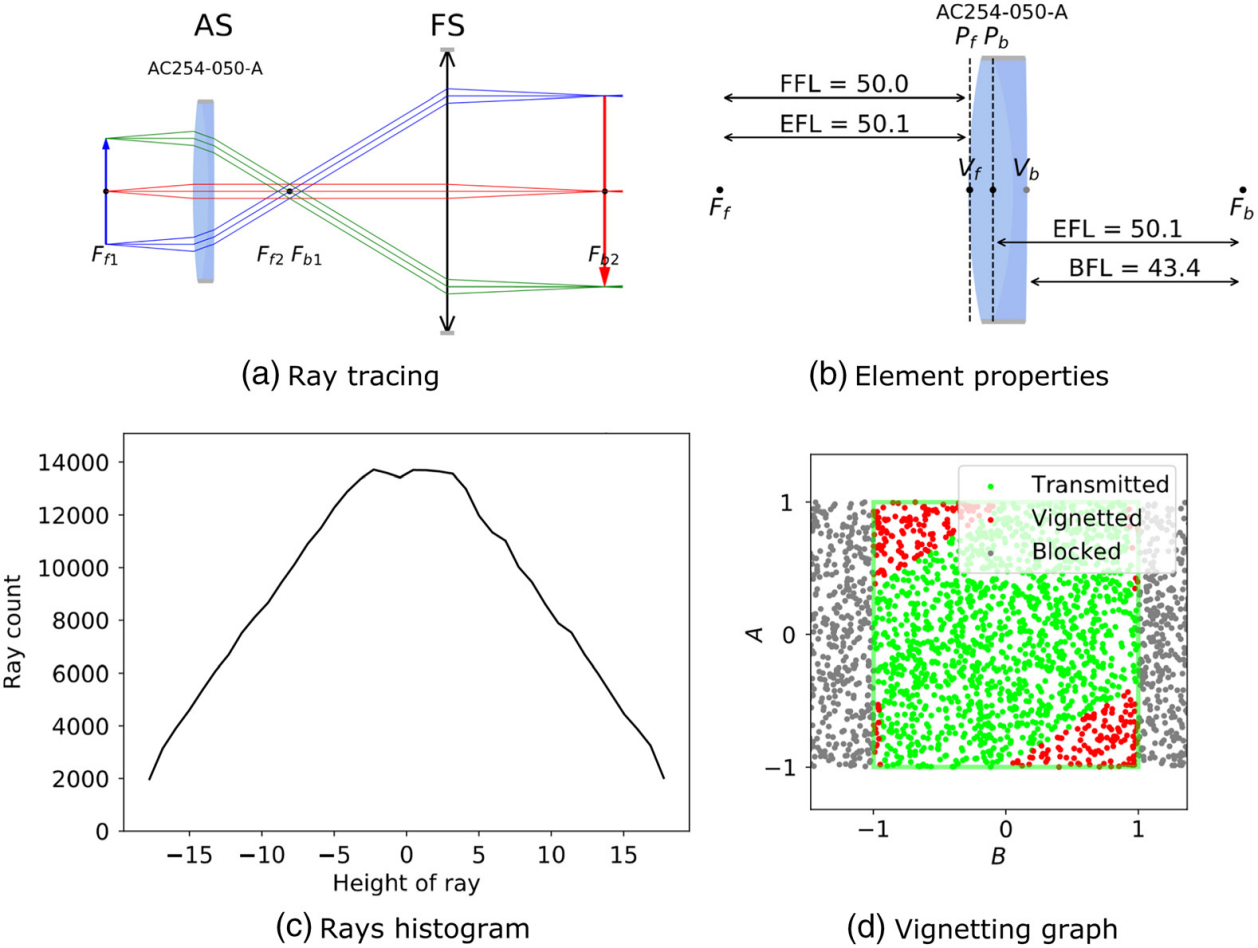
Some of the main features of the raytracing module overview. A 4f-system is defined using a thin lens and a Thorlabs doublet lens for which we (a) trace the imaging path; (b) display element properties for the Thorlabs lens AC-254-050-A where $V_{f,b}$ represent the front and back vertices and $P_{f,b}$ are the front and back principal planes; (c) show the ray count distribution for the rays at the output; and (d) display its vignetting graph.

Four graphical options that convey different and complementary information are available and shown in Fig. 4. As was shown before, once the imaging path is defined, all the components can be depicted using display() [shown in Fig. 4(a)] with default input rays. Figure 4(b) shows that a single component such as a thick lens or a commercial lens can be displayed with the BFL, front focal length (FFL), and any planes of interest. If desired, user-provided rays or ray distributions can be used to compute the intensity profile at any point in an optical system using a Monte Carlo algorithm, as shown in Fig. 4(c). Finally, reportEfficiency() enables the user to study the vignetting caused by the inappropriate diameters of the lenses and apertures in an imaging path [an example is shown in Fig. 4(d)]. These options are discussed next but specific examples are presented in the examples Sec. 6.

#### 5.2.1 Display

Used without any argument on an ImagingPath, display exhibits three ray groups in green, red, and blue, each group containing three rays, with an object corresponding to the FOV of the system as can be seen in Fig. 4(a). It is possible for the user to adjust the height and position of the object, and the number of displayed rays using the convenience function displayWithObject instead of display:

(...)

path.displayWithObject(diameter=5, fanNumber=4, rayNumber=2)

More than one source of rays may be traced using display(raysList=[...]) and adding as many ObjectRays and LampRays as desired [collections of rays with certain properties such as diameter, numerical aperture (NA), and angular spread]. An example of this functionality is presented in Sec. 6.2. Used on a component (e.g., thorlabs.AC254_050_A().display()), it will show the important properties.

#### 5.2.2 Ray collections and histograms

The relative output intensity can be obtained by tracing a collection of rays with a given angular distribution, such as a random uniform distribution or a random Lambertian distribution.^14^ These rays can then be traced through an optical system to obtain the output distribution using traceManyThrough() or traceThrough(), with the following code (Sec. 6.3 show an example):

*# RandomUniformRays Example*

**from** raytracing **import** *

*# define a list of rays with uniform distribution*

inputRays = RandomUniformRays(yMin = -5,

yMax = 5,

maxCount = 1000000,

thetaMax = 0.5,

thetaMin = -0.5)

inputRays.display()

*# Define path elsewhere*

outputRays = path.traceManyTrough(inputRays)

outputRays.display()

It is worth mentioning that since the rays are randomly emitted with RandomUniformRays() and RandomLambertianRays(), the results and profiles will slightly change every time the code is run.

#### 5.2.3 Efficiency and vignetting

Displaying the system helps identify the position of the apertures but it is often necessary to better quantify the design in addition to visualizing it. The module makes use of the fact that any ray in the system can be represented by a linear combination of two other rays 1 and 2:

$$y_{3}=\frac{I_{32}}{I_{21}}y_{1}+\frac{I_{13}}{I_{21}}y_{2},$$ (7)

$$\theta_{3}=\frac{I_{32}}{I_{21}}\theta_{1}+\frac{I_{13}}{I_{21}}\theta_{2},$$ (8)

where $I_{ij}$ is the Lagrange invariant between ray i and j [Eq. (6)]. When rays 1 and 2 are the principal and axial rays, the coefficients $A=\frac{I_{32}}{H}$ and $B=\frac{I_{13}}{H}$ are of great importance, because their values can be used to determine if a ray is vignetted or simply blocked, identifying the element causing vignetting in a system. Indeed, if A and B are higher than one for a certain ray, it is expected that this ray is blocked. If they are less than one, but the ray is still blocked, it means an element in the system causes vignetting, therefore limiting the invariant. In the raytracing module, using reportEfficiency() on an ImagingPath presents all of those characteristics and even more, such as the size of the FOV, the position of the element causing vignetting, and the NA at the object plane. A more complete example of using the invariant is described in Sec. 6.

## 6 Practical Examples Solved with Module

### 6.1 Confocal Laser Scanning Microscope

**Fig. 5:**
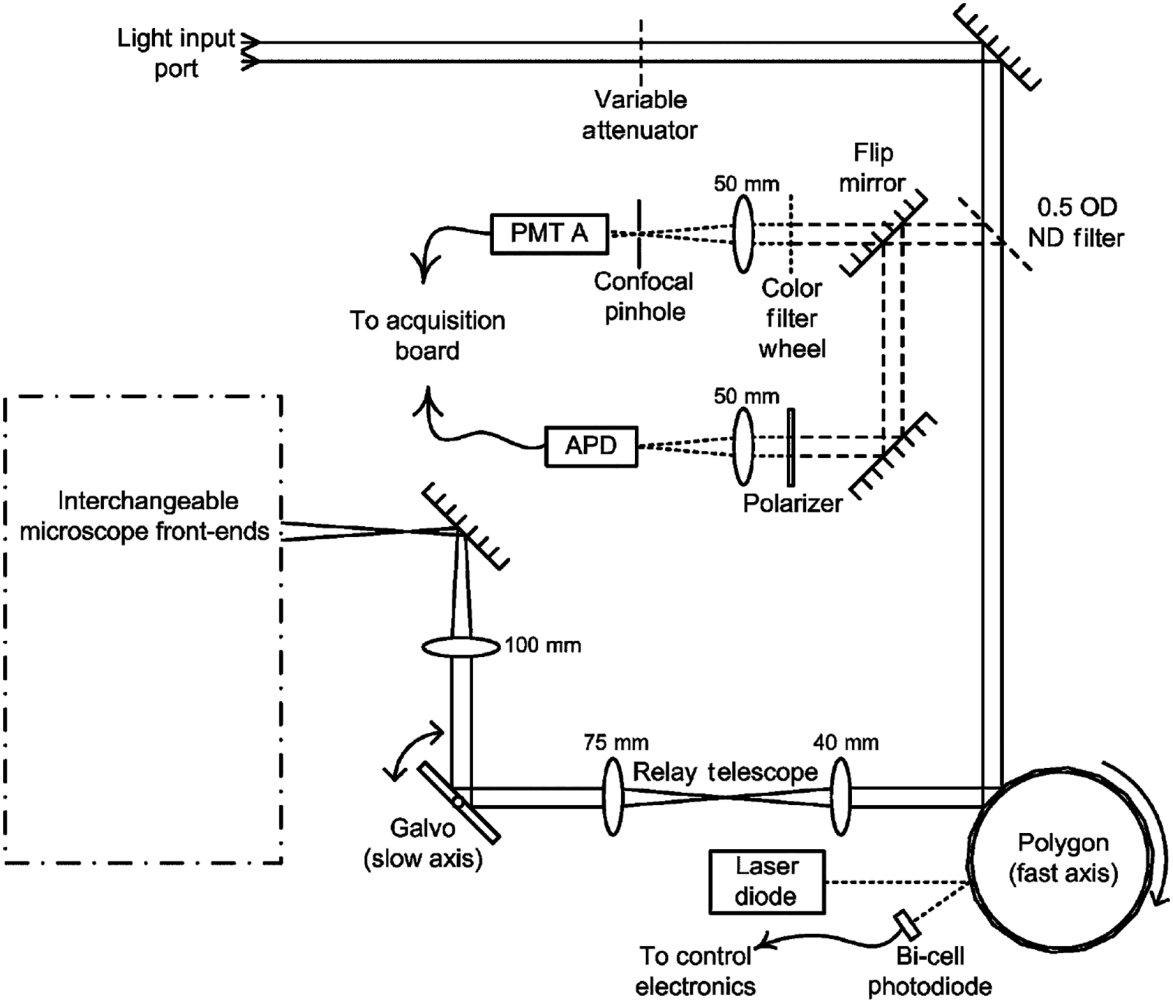
CLSM platform and optical design: In this CLSM, the spinning polygonal mirror and the galvanometer scanner mirror perform raster scanning at the sample and the 4f systems are used to relay the light reflected from the scanning mirror to the sample.^16^

**Fig. 6:**
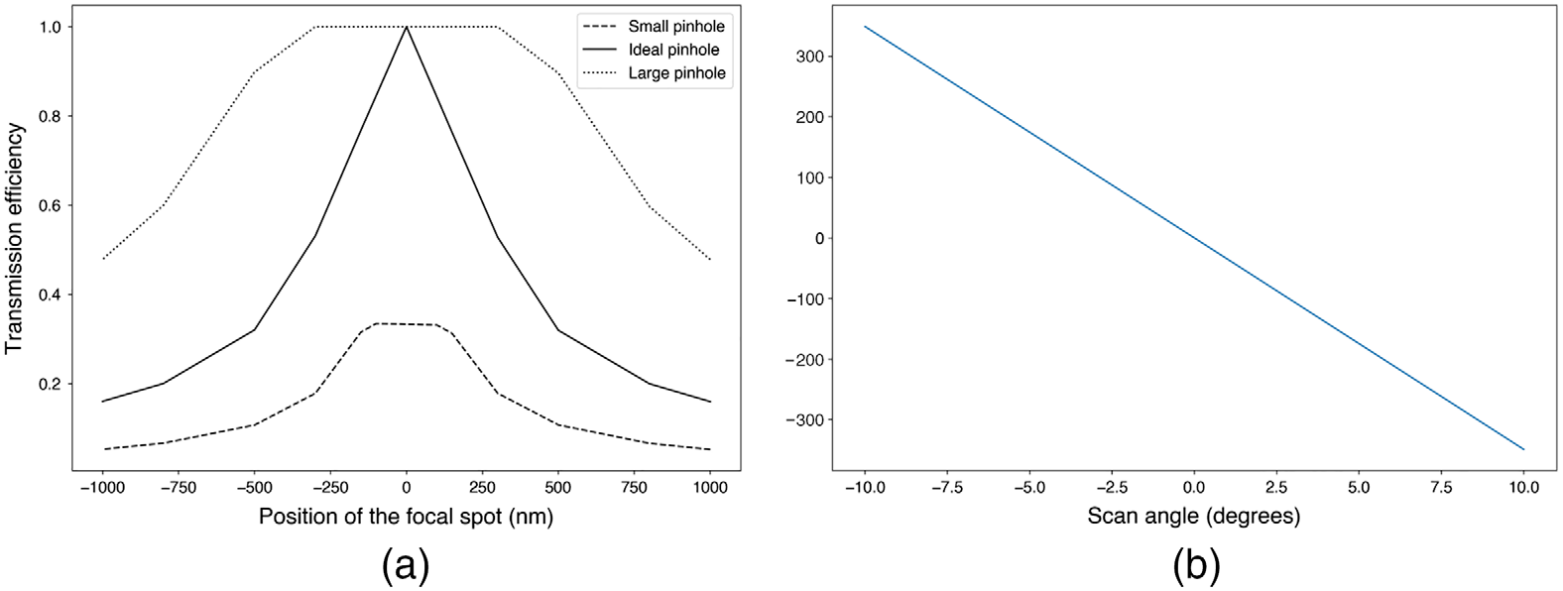
(a) Transmission efficiency according to the position in depth relative to the ideal position (0) for three different pinhole sizes. (b) Graph showing scanning position according to the scan angle of the polygon mirror.

The main components of any confocal laser scanning microscope (CLSM) are a pinhole, conjugated to the illumination focal spot to block the out of focus light, and a set of mirrors to scan the laser beam across the sample.^15^ Ideally, the pinhole matches the size of the focal spot at the sample to produce appropriate optical sectioning.^15^ The scanning mirrors are at the focus of the scan lens to create a raster scan at the object. We can calculate optical sectioning as a function of pinhole size and show the covered FOV by the scanning components of the following CLSM^16^ (Fig. 5). At first, the importance of the appropriate pinhole size in the final image formation is shown by sending a large number of rays in the optical system and computing how many make it through the pinhole for different focal spot positions. The output is a graph that presents the transmission efficiency, i.e., the number of output rays at the pinhole, divided by the number of input rays, as a function of the position of the focal spot (Fig. 6). The solid curve in Fig. 6(a) shows that, with the ideal pinhole size, the only position at which the transmission efficiency is optimal is at the focal plane of the objective, yielding optimal sectioning.

Moreover, the covered horizontal FOV of the polygonal mirror according to the scan angle at the polygon plane is determined using the raytracing module, as it is shown in Fig. 6(b). In this part of the example, the object is considered to be the laser beam at the polygonal mirror plane. Therefore, only the path from the polygonal mirror to the sample is considered. Figure 6(b) shows that the polygon mirror produces a straight line of ≈704  nm of length in that optical system using the scan angle between 10° and −10° at the polygon plane. It is possible to use the same method to determine the vertical FOV using the scan angle of the galvo mirror. The code related to the optimal pinhole size and scanning position has been written in Sec. 9.1.

### 6.2 Köhler Illumination

**Fig. 7:**
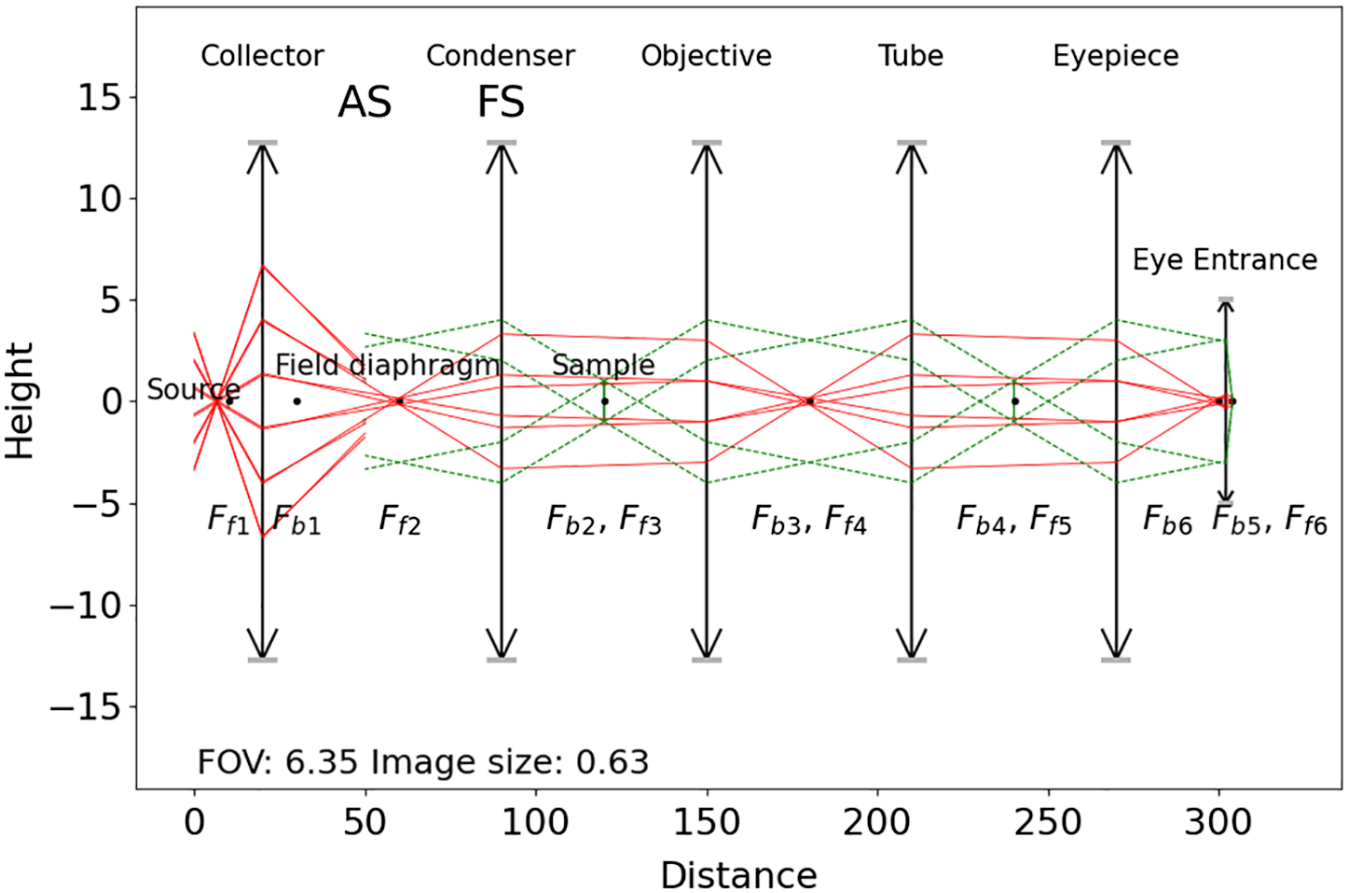
Scheme of a conventional widefield microscope with the light source path (red) and the sample path (dashed green) with a Kohler illumination.

The purpose of Köhler illumination is to provide a uniform light intensity at the sample.^15^ The opposite illumination system is called critical illumination whereby the light source is imaged onto the specimen.^15^ The main element to design Köhler illumination is an extra lens, i.e., the collector lens, close to the light source, which leads to uniformity by way of imaging the Fourier transform of the light source profile onto the specimen instead of the light source profile itself. This example verifies the design of Köhler illumination with the raytracing module, as it is shown in Fig. 7. One can see that the conjugate planes of the light source do not overlay with the conjugate planes of the sample, showing that the light source is not imaged on the object and the object is also illuminated with parallel uniform light. The code of this example is available in Sec. 8.2.

### 6.3 Widefield and Multiphoton Microscopy

As presented in Secs. 3 and 5, the Lagrange invariant, H, is a constant related to the collection efficiency of an optical system and can be used to find the limiting element(s) in an optical system.^12^ The locations of AS and FS in optical systems, such as a widefield microscope^15^ or multiphoton microscope,^17^ allow us to validate their designs. Optimizing both systems is possible with the raytracing module: an example of a widefield microscope is provided in this section while an example of a multiphoton microscope showing the detector size importance in its design is provided in (https://github.com/DCC-Lab/RayTracing/tree/master/examples). The example presents different optical systems in which AS and FS are in different positions using different sizes of lenses. This example shows how the locations of AS and FS, determined by the size of the lenses, affect the number of rays that can reach the image plane.

**Fig. 8:**
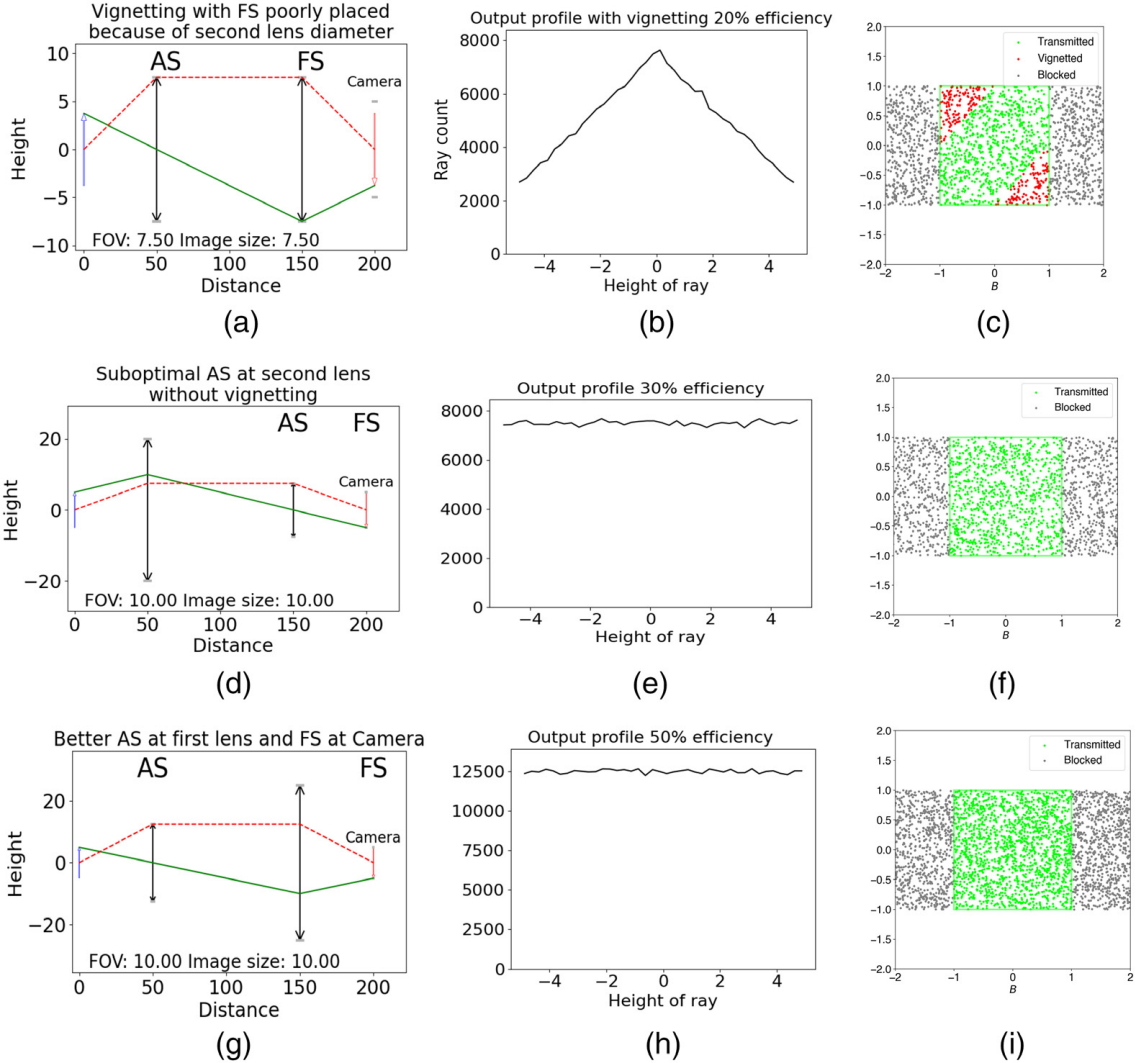
(a) The 4f system where $d_{1}=d_{2}$. Both lenses are too small, so the AS is at the first lens and the FS is at the second lens, so not all of the object is going to be imaged. (b) Transmission efficiency according to the height of the detected ray; (c) the corresponding vignetting plot. The red dots in the figure represent vignetting happened by poorly placed FS. (d) 4f system where $d_{1}>d_{2}$ Thus, the AS is at the second lens and FS is at the camera. (e) The transmission efficiency according to the height of the detected ray and (f) the corresponding vignetting plot. There is no vignetting in this optical system, but the number of transmitted rays is low because of the suboptimal AS at the second lens. (g) The 4f system where $d_{1}<d_{2}$. Both lenses are big enough, so the first lens is the AS and the camera is the FS. (h) The transmission efficiency according to the height of the detected ray and (i) the corresponding vignetting plot. The number of transmitted rays is high contrary to the Fig. 8(f) because of the better positioning of the AS and FS.

As it is shown in Fig. 8(a), AS is at the first lens and FS is at the second one. Both lenses in this system are the same and too small, so the principal ray (green line) does not start at the top edge of the object and not all of the object is going to be imaged. Therefore, the output profile in Fig. 8(b) includes vignetting at the image plane. The presence of red dots on the vignetting plot of Fig. 8(c) verifies this too. Figure 8(d) shows a similar 4f system while the second lens is smaller than the first one. AS is at the second lens and FS is at the camera. The output profile in Figs. 8(b) and 8(e) show that there is no vignetting at the image plane, but the small number of rays reaching the image plane at each height could be improved by optimizing the location of the AS in the system.

Finally, the last optical path in Fig. 8(g) contains two different lenses where the first one is smaller. The first lens is AS and the camera is FS. The output profile in Fig. 8(h) shows that more than 12,000 rays are detected at each height at the image plane compared to 8000 in Fig. 8(b). In addition, there are much more green dots present in Fig. 8(i) showing that more rays are detected at the image plane and verifying its assembly. The code of this example is available in Sec. 9.3.

## 7 Discussion and Outlook

This tutorial presents the main features of the ray matrix formalism, in combination with a useful Python module to help visualize optical systems and to understand how they behave within the paraxial approximation. Although the paraxial approximation (sin θ≈θ) introduced early in the formalism may appear unnecessary at first sight, it is, in fact, essential. Unbeknown to many of us, the exact vector expression to calculate a refracted ray from an incident ray at any interface is nonlinear in the cosine of the incident angle.^11^ The paraxial approximation provides a simpler linear description of rays and optical elements that makes discussions of designs easier by relying on only a few simple concepts, all while being valid close to the axis even in complex systems. In addition, the transfer matrix method central to this formalism becomes very convenient to study general behaviors without focusing too much on the details. On the other hand, without the paraxial approximation, many terms such as focal planes, cardinal points, or magnification cease to be well-defined concepts. Another consequence is that the description of optical elements interfaces would now require a refined description of the surface profile beyond the simple spherical approximation, which may not always be available, and disregarding this detail would defeat the purpose of neglecting the paraxial approximation in the first place. For this reason, the ray matrix formalism always makes use of the paraxial approximation. See the very complete and very approachable book by Kloos for an excellent discussion of its necessity in its introductory chapter.^11^

This Python module can be used to design and optimize a system before building it, but it can also be used to validate an already built optical system. To build a realistic system, many optical elements included in the module (e.g., simple and complex lenses, dielectric interface but also commercial lenses) can be combined to model the system under study. The ABCD transfer matrix for each element describes the transformation of a ray between two reference planes.^6^ Common properties of the system such as focal distances, conjugate planes, magnification, and principal planes are provided by methods in this object-oriented design.

Apertures are not formally included in the ray matrix formalism because the blocking of a ray is not a linear transformation that can be expressed with a matrix transformation. Indeed, the apertures in the imaging path limit the image size and the amount of light that reaches the image plane. Therefore, it is important to consider them when modeling and building optical systems. With the simple addition of apertures with ray tracing, a wealth of information about the system becomes available, most importantly AS and FS. Although finding AS and FS in simple systems is straightforward, the presence of multiple lenses in a system makes it challenging to find their location without a noticeable amount of tedious calculations. The raytracing module simplifies the calculation of the positions and diameters of AS and FS. The impact of vignetting on the output profile can also be evaluated to help identify the limiting apertures, opening the door to further optimization.

Although the given examples are intended to be inclusive, many other things can be done: it is possible to extract the final wavefront of a beam originating from a given point or to perform resonator studies with Gaussian beams since they can be modeled with the same formalism.^18^ All the functions and their details (API reference) are accessible to all users online at Ref. 3. Below is a list of key classes and useful functions to help getting started with raytracing (Tables 1 and 2). In addition, many well-known lenses and objectives from Thorlabs, Edmund Optics, Nikon, and Olympus or special elements such as an axicon with its apex angle are defined and can be used whenever needed.

**Table 1:** Useful classes of the raytracing module.

Classes	Descriptions
Ray()	A light ray as transformed by ABCD matrices
Rays()	A group of rays
Matrix()	General ABCD matrix from which all elements are derived
Aperture()	An aperture of finite diameter
Lens()	A thin lens of focal f
Space()	Free space of length d
ImagingPath()	Sequence of ABCD matrices with an object at the front
RandomUniformRays()	A list of random rays with Uniform distribution
RandomLambertianRays()	A list of random rays with Lambertian distribution
GaussianBeam()	A coherent laser beam

**Table 2:** Useful functions of the raytracing module.

Classes	Functions	Descriptions
Matrix()	effectiveFocalLengths()	The effective focal length
	magnification()	The magnification of the element
	trace()	The traced ray through an optical system
ImagingPath()	apertureStop()	Position and diameter of AS
	fieldStop()	Position and diameter of FS
	lagrangeInvariant()	The Lagrange invariant
	axialRay()	Axial ray of the system
	chiefRay()	Chief ray of the system
	reportEfficiency()	Efficiency report for the system

The raytracing module is developed to facilitate the simulation of an optical path for non-experts, however, there are some limitations. The use of this matrix-based formalism is advantageous compared to other calculation strategies such as diffraction integrals with finite element modeling. However, the simulations based on the finite difference time domain simulations, finite element modeling, or other numerical solver algorithms are time-consuming and they require high memory and processing power. In contrast, this module uses matrix calculations that are extremely fast but are limited to the first-order paraxial approximation and do not consider diffraction, geometrical aberrations, or interference. The results thus obtained must therefore be considered within these limitations. Finally, unlike commercial packages such as Zemax, Oslo, and Code V, the raytracing module does not include embedded optimization algorithms. However, the user is free to sweep through the parameters to perform optimizations manually.

There are many other things that could be added to the raytracing module such as the complete 2D calculations for rays in the transverse plane, tilted optical components and even misaligned optical elements that could be treated with an expansion of the formalism (e.g., ABCDEF matrices). The possibility of reading files from commercial packages such as Zemax would also be welcome, but recent formats are not always publicly documented. Also, adding an optimization package with a few parameters is one possible area for further development, a general-purpose algorithm appears non trivial at this point. However, the goal is not to be complete, but rather to be useful and simple to use for non-experts. The code being Open Source, contributions (e.g., more commercial components, fixing issues, and suggestions.) can be made through the raytracing GitHub page at (https://github.com/DCC-Lab/RayTracing).

## 8 Conclusion

The goal of this paper is to introduce the raytracing module, a freely available, well-developed simple tool for designing, optimizing, and quantifying optical systems by calculating the optical properties based on ABCD transfer matrices of the elements (version 1.3.0 corresponds exactly to this paper). Since it does not have the complexity of some advanced optical software, it can be used by beginners and students in the field of optical design. We emphasize once more in closing that the two main features that make this module convenient are the consideration of physical apertures and the freedom to use any desired light source for simple practical calculations.

## 9 Code

The code for the examples of Sec. 6 is available here.

### 9.1 Confocal Laser Scanning Microscope Example

This is the code used in the CLSM example in Sec. 6.1.

**import** envexamples

**from** raytracing **import** *

**import** matplotlib.pyplot as plt

**import** numpy as np

thetas = []

positions1 = []

objectHalfHeight =focalRadius= 0.000250

scanAngle = 10*np.pi/180

pinholeModifier = **{**1 / 3: [], 1: [], 3: []**}**

positions = [1000, 800, 500, 300, 150, 100, 50, 25, 0, -25, -50, -100, -150, -300, -500, -800, -1000]

nRays = 100000

scanRays = UniformRays(yMax=0, thetaMax=scanAngle, M=1, N=nRays)

inputRays = RandomUniformRays(yMax=focalRadius, yMin=-focalRadius, maxCount=nRays)

focalSpotPosition=objFocalLength = 5

**class** UISUPLAPO60XW(Objective):

 **def** __init__(self):

**super**(UISUPLAPO60XW, self).__init__(f=180/60,

NA=1.2,

focusToFocusLength=40,

backAperture=7,

workingDistance=0.28,

magnification=60,

fieldNumber=22,

label=’UISUPLAPO60XW Objective’)

**def** illuminationPath1():

 illumination1 = ImagingPath()

 *# The object in this situation is the laser beam at the scanning element.*

 illumination1.objectHeight = objectHalfHeight*2

 illumination1.rayNumber = 3

 illumination1.fanNumber = 3

 illumination1.fanAngle = 0

 illumination1.append(System4f(f1=40, f2=75, diameter1=24.5, diameter2=24.5))

 illumination1.append(System4f(f1=100, f2=100, diameter1=24.5, diameter2=24.5)) illumination1.append(Space(d=180/40))

 illumination1.append(UISUPLAPO60XW())

 illumination1.append(Space(d=180/40))

 **return** illumination1

path1 = illuminationPath1()

outputRays1 = path1.traceManyThrough(scanRays)

**for** i **in range**(len(outputRays1)):

thetas.append(scanRays[i].theta*180/np.pi)

positions1.append(outputRays1[i].y*1000)

scanRays.displayProgress()

plt.plot(thetas,positions1)

plt.xlabel(’Scan angle (degrees)’, fontsize=20)

plt.ylabel(’Scanning position of the focal spot ($\micro$m)’, fontsize=20)

plt.show()

#--------------------------------------------------

**def** path(focalSpotPosition=objFocalLength):

illumination2 = ImagingPath()

illumination2.append(Space(d=focalSpotPosition))

illumination2.append(Lens(f=objFocalLength))

illumination2.append(Space(d=105))

illumination2.append(Lens(f=100))

illumination2.append(Space(d=100))

illumination2.append(System4f(f1=100, f2=75))

illumination2.append(System4f(f1=40, f2=50)) # *Path finishes at the pinhole position*

 **return** illumination2

**def** optimalPinholeSize():

 """

 *Finds the magnification of the optical path and use it to find the optimal pinhole size when the focal spot is at one*

 *focal length distance of the objective.*

 *Return*

 -----------

  *pinholeIdeal : Float*

   *Returns the optimal pinhole size*

 """

 # *Dictionnary of the position and magnification of all conjugate planes of the focal spot.*

 planes = path().intermediateConjugates()

 # *The last conjugate plane is the pinhole. The magnification of this position is saved in mag.*

 mag = planes[-1][1]

 # *Calculates the pinhole size that fits perfectly the focal spot diameter.*

 pinholeIdeal = abs(mag * (focalRadius * 2))

 **return** pinholeIdeal

**def** rayEfficiency(pinholeFactor=None, focalSpotPosition2=None):

 """

 *Determines the amount of rays emitted from the object that are detected at the pinhole plane.*

 *Parameter*

 #---------------

  *pinholeFactor : Float*

   *Factor changing the pinhole size according to the ideal pinhole size.*

  *focalSpotPosition : float*

   *Position of the focal spot according to the objective (first lens)*

 *Returns*

 ------------

  *illumination : object of ImagingPath class.*

   *Returns the illumination path*

 """

 illumination2 = path(focalSpotPosition2)

 pinholeSize = optimalPinholeSize() * pinholeFactor

 illumination2.append(Aperture(diameter=pinholeSize))

 # *Counts how many rays make it through the pinhole*

 outputRays2 = illumination2.traceManyThroughInParallel(inputRays, progress=False)

 **return** outputRays2.count / inputRays.count

**for** pinhole **in** pinholeModifier:

 **print**("\nComputing transmission for pinhole size **{**0:0.1f**}**".**format**(pinhole))

 efficiencyValues = []

 **for** z **in** positions:

  **print**(".",end='')

  newPosition = 5 + (z * 0.000001)

  efficiency = rayEfficiency(pinholeFactor=pinhole,   focalSpotPosition2=newPosition)

 efficiencyValues.append(efficiency)

 pinholeModifier[pinhole] = efficiencyValues

 plt.plot(positions, pinholeModifier[1 / 3], ’k:’, label=’Small pinhole’, linestyle=’dashed’)

 plt.plot(positions, pinholeModifier[1], ’k-’, label=’Ideal pinhole’)

plt.plot(positions, pinholeModifier[3], ’k--’, label=’Large pinhole’, linestyle=’dotted’)

plt.ylabel(’Transmission efficiency’, fontsize=20)

plt.xlabel(’Position of the focal spot (nm)’, fontsize=20)

plt.legend()

plt.show()

### 9.2 Kohler Illumination System Example

**from** raytracing **import** *

illumination = ImagingPath()

illumination.design(fontScale=1.5)

illumination.append(Space(d=20))

illumination.append(Lens(f=10, diameter=25.4, label="Collector"))

illumination.append(Space(d=30))

illumination.append(Aperture(diameter=2, label="Field diaphragm"))

illumination.append(Space(d=10+30))

illumination.append(Lens(f=30, diameter=25.4, label="Condenser"))

illumination.append(Space(d=30+30))

illumination.append(Lens(f=30, diameter=25.4, label="Objective"))

illumination.append(Space(d=30+30))

illumination.append(Lens(f=30, diameter=25.4, label="Tube"))

illumination.append(Space(d=30+30))

illumination.append(Lens(f=30, diameter=25.4, label="Eyepiece"))

illumination.append(Space(d=30+2))

illumination.append(Lens(f=2, diameter=10, label="Eye Entrance"))

illumination.append(Space(d=2))

illumination.display(interactive=False, raysList=[LampRays(diameter=0.1, NA=0.5, N=2, T=6, z=6.6666666, rayColors=’r’, label="Source"),ObjectRays(diameter=2, halfAngle=0.1, H=2, T=2, z=120, rayColors=’g’, color=’g’, label="Sample")], removeBlocked=False)

### 9.3 Widefield Microscope Example

The code for the example 6.3 is written below.

**from** raytracing **import** *

*# Defines the path. a and b are the diameter of the lenses.*

**def** imagingPath(a=10, b=10, title=""):

path = ImagingPath()

path.label=title

path.append(System4f(f1=50, diameter1=a, f2=50, diameter2=b))

path.append(Aperture(diameter=10, label=’Camera’))

**return** path

*# Input from the expected field of view*

nRays=1000000

objectHalfHeight = 5

inputRays = RandomUniformRays(yMax = objectHalfHeight,yMin = -objectHalfHeight,thetaMin = -0.5, thetaMax = +0.5,maxCount=nRays)

*# Three paths with different sets of lens diameter.*

path1 = imagingPath(a=d1, b=d2, title="Vignetting with FS poorly placed because of second lens diameter")

outputRays = path1.traceManyThrough(inputRays)

efficiency = 100*outputRays.count/inputRays.count

path1.display(limitObjectToFieldOfView=False, onlyChiefAndMarginalRays=True)

outputRays.display("Output profile with vignetting **{**0:.0f**}**\% efficiency".**format**(efficiency), showTheta=False)

path1.reportEfficiency()

path2 = (...) *# same as path1*

path3 = (...) *# same as path1*
